# Fabrication and Anti-Swelling Properties of Gelatin/Sodium Alginate–Carboxymethyl Chitosan-Based Cationic Coordination Hydrogels

**DOI:** 10.3390/foods14183149

**Published:** 2025-09-09

**Authors:** Haixin Zhao, Jinlong Li, Shuang Cong, Hongman Hou, Gongliang Zhang, Jingran Bi

**Affiliations:** State Key Laboratory of Marine Food Processing & Safety Control, Dalian Polytechnic University, No. 1, Qinggongyuan, Ganjingzi District, Dalian 116034, China; zhx_010822@126.com (H.Z.);

**Keywords:** cationic coordination, polyelectrolyte interaction, pH-responsive, anti-swelling, pH-responsive release

## Abstract

In this study, the effect of cationic participation on the swelling behavior and pH-responsive release characteristics of polyelectrolyte hydrogel based on gelatin (Gel), sodium alginate (Alg), and carboxymethyl chitosan (CMCS) was explored. The shell–core morphology of the cationic coordination hydrogels was prepared by introducing Na^+^, Ca^2+^, and Fe^3+^ into the crosslinking system, which significantly altered the inherent pH-responsive swelling properties of Gel/Alg-CMCS hydrogel. The modified hydrogel demonstrated a release resistance of carvacrol (CAR) under acidic conditions while facilitating rapid release under neutral conditions. Notably, the CAR release profile was substantially modified by the distinct anti-swelling properties of cationic coordination hydrogels. In particular, Gel/Alg-CMCS-Fe^3+^ hydrogel exhibited high accumulative release of 58.34% at pH 1.0 while maintaining a minimal release degree of merely 7% in weakly acidic and neutral environments. These intriguing findings provide valuable insights into intelligent active delivery for future applications.

## 1. Introduction

pH-responsive hydrogels serve as crucial delivery challenge systems for addressing various fields due to their unique environmental sensing capabilities. In the food industry, pH-responsive hydrogels can protect flavor compounds and functional ingredients (e.g., vitamins, polyphenols) by initiating a controlled release mechanism in response to specific pH levels [1,2,3]. This targeted release significantly enhances product stability and functional properties. To address the volatilization and inactivation of bioactive compounds, particularly labile flavor compounds during processing, storage, and digestion, pH-responsive hydrogels achieve precise encapsulation and sustained release delivery through dynamic responses to endogenous pH changes in food matrices. In the field of active substance delivery, pH-responsive hydrogels extend their advantages to complex physiological environments by responding to the pH gradient along the digestive tract (oral pH 6.5–7.5, gastric pH 1.0–3.0, intestinal pH 6.0–7.4) [4]. They overcome key limitations of conventional oral formulations—including degradation by gastric acid, inadequate intestinal absorption, and poor targeting—while enabling efficient active substance delivery following oral administration, facilitating absorption in the stomach [5,6]. This capability supports applications in functional foods, such as formulations containing digestive enzymes or iron agents. From the perspectives of stabilizing food components, regulating processing textures to delivering across biological barriers, the application requirements for pH-responsive hydrogels now span the entire chain from agricultural production to food processing and human health.

Carvacrol (CAR), a phenolic compound found in oregano and thyme, possesses potent antioxidant activity and is commonly used in food applications due to its flavor and aroma, as well as its ability to inhibit oxidation and extend shelf life. Its application is limited by low water solubility, poor stability, high volatility, and susceptibility to degradation under acidic conditions. However, these limitations also make it an ideal model compound for studying pH-responsive flavor-controlled release. It precisely aligns with the requirements of food processing (protection in acidic environments) and oral/intestinal delivery (targeted release in neutral conditions) while also providing a universal platform for the controlled release of other volatile active substances (e.g., limonene, menthol). Cheng et al. [7] prepared a pH-responsive carboxymethyl chitosan/sodium alginate/carvacrol hydrogel by hydrogen bonds and ion–dipole interaction.

Polyelectrolyte interaction is a common method for forming pH-responsive hydrogels, whose principle is based on pH changes inducing alterations in the solubility of matrix materials or internal molecular crosslinking to form the hydrogel. Zhang et al. [8] prepared a double-crosslinked sodium alginate/chitosan hydrogel with pH responsiveness and good stability through electrostatic interactions between oppositely charged polysaccharides, which can protect fruit from ripening, dehydration, and microbial invasion. Khan et al. [9] developed a sulfur-functionalized chitin composite chitosan/gelatin film with excellent antioxidant and antibacterial properties, good mechanical properties, and physicochemical characteristics. Xu et al. [10] fabricated a pH-responsive manganese-based carboxymethyl chitosan hydrogel for pesticide release. Zhang et al. [11] prepared a novel pH-responsive nanohydrogel using o-carboxymethyl chitosan and sodium alginate. o-Carboxymethyl chitosan, as a cationic polysaccharide, has -NH_2_ groups that can be protonated to -NH_3_^+^, undergoing polyelectrolyte interactions with -COO^−^ dissociated from the carboxyl groups of the anionic polysaccharide sodium alginate to form a network structure.

Owing to their stability, high water absorption, water retention, and swelling properties [12], hydrogels can absorb water hundreds of times their own weight. However, excessive swelling can lead to the destruction of the molecular chain structure, a decline in mechanical properties, an increase in the degradation rate, and limited usability. To address these issues, researchers have proposed the concept of anti-swelling hydrogels [13], which aim to control the swelling ratio of hydrogels by regulating the molecular pore structure and crosslinking degree, thereby enhancing their performance. The network structure and chemical composition of hydrogels are key factors influencing their swelling behavior. Effective strategies for suppressing hydrogel swelling include introducing hydrophobic monomers or grafting hydrophobic side groups onto hydrophilic backbones, constructing hydrophobic crosslinking junctions, increasing the crosslinking density of the hydrogel network, and avoiding or minimizing the incorporation of ionic monomers [14,15]. For instance, Di et al. [16] developed double-network (DN) hydrogels using a combined physical/chemical crosslinking approach. By immersing the hydrogel in an iron (Fe^3+^) solution, a transition-state double crosslinked coordination hydrogel was formed. The introduction of hydrophobic monomers and hydrogen bonding further endowed the hydrogel with excellent anti-swelling properties. Similarly, Xu et al. fabricated a homogeneous anti-swelling hydrogel through a solvent exchange strategy involving dimethyl sulfoxide (DMSO) [17]. The incorporation of DMSO improved the conformational uniformity of the polymers, resulting in hydrogels with superior rigidity, toughness, and anti-swelling properties. However, this approach also limited the in vivo applications of the hydrogels. With the advancement of anti-swelling hydrogels, their potential in biomedical fields, such as tissue engineering, biosensors, and drug delivery systems, continues to expand. Li et al. [18] employed a “one-step immersion method” to prepare Fe^3+^ dual-crosslinked sodium alginate/poly(acrylamide–acrylic acid) copolymer double-network hydrogels. These hydrogels achieve high strength and toughness through the strong coordination capacity between abundant carboxyl groups on alginate molecules and copolymer chains with multivalent metal ions. Li et al. also investigated the effects of other cations (Ag^+^, Na^+^, Ca^2+^, Cu^2+^, and Al^3+^) on their mechanical properties. However, none of the hydrogels prepared with these five cations exhibited superior performance compared to those containing Fe^3+^. Zhang et al. [19] developed polyvinyl alcohol–chitosan/sodium alginate–Ca^2+^ hydrogels with a core–shell structure to study the factors influencing their swelling properties. Cu^2+^ imparts a blue color to hydrogels and possesses certain biological toxicity, making it highly unsuitable for food or oral drug applications. Mg^2+^ has a small ionic radius but a large hydrated ionic radius and high charge density, resulting in weak binding capacity with polymers and difficulty in forming stable structures, ultimately yielding softer gels. Luo et al. [20] immersed chitosan (CS)/polyvinyl alcohol (PVA) hydrogels in NaCl solution, enhancing their mechanical properties and imparting anti-swelling characteristics. Overall, the anti-swelling hydrogels fabricated via ionic coordination crosslinking exhibit exceptionally high mechanical strength and anti-swelling properties, while the coordination bonds are regulated by environmental pH, enabling the precise on-demand release of active substances. Thus, we selected Na^+^, Ca^2+^, and Fe^3+^ to crosslink with hydrogels, constructing a research system ranging from weak to strong and from simple to complex in terms of performance and mechanisms. The fundamental principle follows the rule that higher valence, greater charge density, and stronger coordination capacity lead to enhanced anti-swelling properties and mechanical strength.

To explore the impact of cationic coordination on anti-swelling and controlling release properties, Na^+^, Ca^2+^, and Fe^3+^ were introduced into the crosslinking system of Gel/Alg-CMCS hydrogel. The unique shell–core morphology of the cationic coordination hydrogel was recorded using scanning electron microscopy (SEM), and its crosslinking mechanism was investigated via Fourier transform infrared spectroscopy (FTIR). The swelling properties of Gel/Alg-CMCS hydrogel and the anti-swelling role of cationic coordination were analyzed. Meanwhile, positive ion also converted the native CAR pH-responsive release behavior of Gel/Alg-CMCS hydrogel, and their behavior was specifically determined. This paper proposes a new strategy for the design of pH-responsive active delivery systems.

## 2. Materials and Methods

### 2.1. Materials

Gelatin (Gel; lot number: 9000-70-8; molecular weight; MW: 10–70 kDa), sodium chloride (NaCl), calcium chloride (CaCl_2_), and ferric chloride (FeCl_3_) were obtained from Aladdin Biotechnology Co., Ltd. (Shanghai, China). Sodium alginate (Alg; lot number: 9005-38-3; MW: 200 kDa), carboxymethyl chitosan (CMCS; lot number: 83512-85-0; MW: 100,000–200,000 Da; degree of carboxylation ≥ 80%), and carvacrol (CAR; lot number: 499-75-2; density: 0.98 g/cm^3^) were purchased from Macklin Biochemical Co., Ltd. (Shanghai, China). All other chemicals and reagents used in this study were of analytical grade.

### 2.2. Preparation of Gel/Alg-CMCS-Based Cationic Coordination Hydrogels

Gel was dissolved in deionized water at 60 °C to obtain a final concentration of 0.02 g/mL. A certain mass of Alg was dissolved in the Gel solution to achieve a mass ratio of Gel solution to Alg solution of 1:1, resulting in a Gel/Alg solution. A certain mass of CMCS was dissolved in the Gel/Alg solution to prepare a Gel/Alg-CMCS mixture with a mass ratio of 1:1:2. The mixture was magnetically stirred at 80 °C for 4 h. After acoustic deaeration to remove bubbles, it was left undisturbed at room temperature overnight to form the Gel/Alg-CMCS hydrogel.

The Gel/Alg-CMCS hydrogels were immersed in 0.1 M of NaCl, CaCl_2_, and FeCl_3_ solutions for 6 h to prepare Gel/Alg-CMCS-Na^+^, Gel/Alg-CMCS-Ca^2+^, and Gel/Alg-CMCS-Fe^3+^ hydrogels, respectively.

### 2.3. Physicochemical Characterizations

#### 2.3.1. Morphology

The hydrogels were dehydrated at 40 °C for 6 h, cryo-fixed in liquid nitrogen, and fractured to expose cross-sections. The cross-sectional morphology was characterized using scanning electron microscopy (SEM; JSM-7800F, JEOL, Tokyo, Japan) operating at an acceleration voltage of 5 kV. The microstructure of the hydrogel was characterized using energy dispersive X-ray spectroscopy (EDX). The elemental composition was measured by line scanning measurements with semi-quantitative analysis.

#### 2.3.2. FTIR

The crushed dried hydrogels were thoroughly mixed with potassium bromide (KBr) in a ratio of 1:100 and then compressed into thin flakes. Fourier transform infrared (FTIR) spectra were then recorded using an FTIR spectrometer (Spectrum II, PerkinElmer, Hopkinton, MA, USA) in the range of 4000 to 400 cm^−1^ with a resolution of 4 cm^−1^.

### 2.4. Rheological Measurements

The viscoelastic properties of the Gel/Alg-CMCS hydrogel were characterized using a DHR-2 rheometer (TA Instruments, New Castle, DE, USA). Frequency sweep tests (0.1–100 rad/s) were performed at 0.5% constant strain. All measurements employed a 20 mm aluminum parallel plate geometry with a 0.5 mm gap.

### 2.5. Mechanical Property Test

Compressive properties were evaluated using an E3000LT Micro-fatigue Tester (Instron Inc., University Ave, Norwood, MA, USA). Cylindrical Gel/Alg-CMCS hydrogel samples (height: 10 mm; diameter: 20 mm) were prepared for compressive testing. Tests were conducted at a 10 mm/min loading rate.

### 2.6. pH-Responsive Swelling Test

The hydrogel was weighed (*W_d_*) and immersed in varying pH buffer solutions. After predetermined intervals, samples were removed, the surface-adsorbed solvent was gently wiped off with filter paper, and swollen hydrogels were immediately weighed (*W_t_*) on an electronic balance. The swelling degree of the hydrogels was calculated using Equation (1):

(1) $$\mathrm{swelling}\;\mathrm{degree}\;(\%)\;=\;\frac{W_{t}\;-\;W_{d}}{W_{d}}\times 100\%$$

Here, *W_t_* is the liquid weight absorbed by the hydrogel at time *t*, and *W_d_* is the dry weight of the hydrogel.

### 2.7. Anti-Swelling Stability Test

The Gel/Alg-CMCS-Ca^2+^ and Gel/Alg-CMCS-Fe^3+^ hydrogels after 120 days of sealed storage at room temperature were taken out and subjected again to swelling tests in different pH solutions.

### 2.8. Hemolysis Assay

Fresh blood was collected from healthy adult Kunming mice via orbital bleeding into a 2 mL EP tube. It was centrifuged at 3000 rpm/min for 5 min at 4 °C. The serum was removed, and the lower red blood cell precipitate was diluted with PBS solution. The hydrogel samples were immersed in PBS solution for 24 h, and 0.8 mL of the immersion solution was taken, followed by the addition of 0.2 mL of red blood cells. A total of 0.8 mL of ultrapure water and PBS solution mixed with 0.2 mL of red blood cells were used as the positive control and negative control, respectively. The mixtures were left standing at room temperature for 0.5 h, centrifuged at 3000 rpm/min for 5 min at 4 °C, and the supernatant was taken to measure its UV absorbance at 541 nm and calculate the hemolysis rate of the test group. Hemolysis was calculated using Equation (2).

*Hemolysis* (%) = [(*OD_sample_* − *OD_negative_*)/(*OD_positive_* − *OD_negative_*)] × 100% (2)

where *OD_sample_, OD_negative_*, and *OD_positive_* represent the absorbance of the test sample, negative control (PBS), and positive control (deionized water), respectively, at 541 nm.

### 2.9. Cytotoxicity Study

Cytocompatibility of the hydrogels was evaluated via CCK-8 assay and GES-1 live/dead staining. Material extracts were prepared by incubating sterile samples in Dulbecco’s Modified Eagle Medium (DMEM; BB4126-1, BestBio Biotechnology Co., Ltd., Shanghai, China) at 37 °C for 24 h (100 mL medium/sample). GES-1 cells were plated in 96-well plates (5000 cells/well) and preconditioned for 24 h (37 °C, 5% CO_2_). Test groups received 200 μL of extract-supplemented basal medium, while negative controls contained medium only.

### 2.10. Ion Migration Test

A total of 0.2 g of Gel/Alg-CMCS-Ca^2+^ and Gel/Alg-CMCS-Fe^3+^ hydrogels was immersed in 50 mL of 10% ethanol aqueous solution. The color of the solutions was measured at 6 h, 8 h, 12 h, and 24 h using a colorimeter, and the lightness value (L), redness value (a), and yellowness value (b) of the samples were recorded, respectively. Based on the above measured data, the total color change (ΔE) in the samples was calculated using Equation (3).

(3) $$∆E=\sqrt{{\left(L^{*}-L_{0}^{*}\right)}^{2}+{\left(a^{*}-a_{0}^{*}\right)}^{2}+{\left(b^{*}-b_{0}^{*}\right)}^{2}}$$

where *L*_0_, *a*_0_, and *b*_0_ are the color parameters of the 10% ethanol aqueous solution without immersed samples; *L*, *a*, and *b* are the color parameters of the 10% ethanol aqueous solution during the immersion process.

The final soaking solution was subjected to inductively coupled plasma optical emission spectrometry (ICP-OES) to determine the content of Fe^3+^ and Ca^2+^. The ion leaching rate was calculated using Equation (4).

(4) $$\mathrm{Mass}\;\mathrm{fraction}\;\mathrm{of}\;\mathrm{iron}\;\mathrm{migration}=\frac{C_{x}\times V}{M_{x}}\times 100\%$$

where *C_x_* represents the concentration of the target element in the solution (mg/L), *V* denotes the volume of the solution (L), and *M_x_* represents the weight of the hydrogel (kg).

### 2.11. Loading Efficiency Test

CAR was added to the Gel/Alg-CMCS precursor solution at a concentration of 15 μL/mL and thoroughly mixed. After removing air bubbles via ultrasonication, the mixtures were allowed to form Gel/Alg-CMCS-CAR hydrogels at room temperature overnight.

The Gel/Alg-CMCS-CAR hydrogels were immersed in different salt solutions (0.1 M NaCl, CaCl_2_, FeCl_3_) for 6 h to prepare Gel/Alg-CMCS-Na^+^-CAR, Gel/Alg-CMCS-Ca^2+^-CAR, and Gel/Alg-CMCS-Fe^3+^-CAR hydrogels.

The CAR-loaded hydrogels were sectioned into 6 mm diameter circular slices and immersed in 20 mL of 10% ethanol. After 1 min of shaking, samples were centrifuged (10,000× *g*, 10 min), followed by UV-Vis spectrophotometric measurement at 276 nm. CAR concentrations were determined using the standard curve, with loading efficiency calculated via Equation (5):

(5) $$\mathrm{loading}\;\mathrm{efficiency}\;(\%)=\;\frac{M_{0}\;-{\;C}_{s}\times V}{M_{0}}\times 100\%$$

where *M*_0_ is the initial addition mass of CAR, *C_s_* is the mass concentration of CAR in the supernatant, and *V* is the volume of the supernatant (20 mL).

### 2.12. CAR Release Behavior Test

The square CAR-loaded hydrogels (1 × 1 cm) were immersed in 20 mL pH buffer solutions at 25 °C. At predetermined intervals, 3 mL of supernatant was collected, mixed with 333 μL of ethanol, and replaced with equal-volume fresh pH-matched buffer. After centrifugation, the CAR concentration was determined at 276 nm by UV-Vis spectrophotometry. Cumulative CAR release was calculated using Equation (6), and the release rate was calculated using Equation (7).

(6) $$\mathrm{cumulative}\;\mathrm{release}\;\left(\%\right)=C_{t}\times V_{\mathrm{total}}+\sum_{0}^{t-1}C_{t}\times V_{t}m_{0}\times 100\%$$

Here, *C_t_* is the mass concentration of the solution at time *t* (mg/mL), *V_t_* is the volume of the solution at time *t* (mL), *V_total_* is the total volume of the solution (mL), and *m*_0_ is the total amount of CAR in hydrogel.

(7) $$\mathrm{release}\;\mathrm{rate}\;\left(\%\right)=\frac{M_{t}\;}{M_{\infty }}\times 100\%$$

Here, *M_t_* is the amount of CAR released after time *t*, and *M_∞_* is the amount of CAR released after time *t_∞_* when the concentration of CAR no longer changes.

Additionally, four kinetic models (Higuchi, Korsmeyer–Peppas, Peppas–Sahlin, and Weibull) were employed to analyze and fit the release behavior of CAR under varying pH conditions.

*Higuchi model*: *M_t_/M_∞_* = *K_h_t*^1/2^ (8)

Here, *M_t_* is the cumulative amount of drug released at time *t*, *M_∞_* is the total amount of drug released at infinite time, and *K_h_* represents the Higuchi kinetic constant.

*Korsmeyer-peppas model*: *ln (M_t_/M_∞_)* = *nlnt* + *lnk* (9)

Here, *M_t_* is the cumulative amount of drug released at time *t*, *M_∞_* is the total amount of drug released at infinite time, *k* is the kinetic constant, and *n* is the diffusion exponent.

*Peppas-Sahlin model*: *M_t_/M_∞_* = *k_1_t^m^* + *k_2_t^2m^* (10)

Here, *M_t_* is the cumulative amount of drug released at time *t*, *M_∞_* is the total amount of drug released at infinite time, and *k*_1_, *k*_2_, and *m* are constants. The first term on the right-hand side represents the Fickian diffusional contribution, *F*, whereas the second term is the case-II relaxational contribution, R.

*Weibull model*: *lnln*(1/(1 − *F*)) = *blnt* + *lna* (11)

Here, *F* and *t* are the cumulative release and time, respectively; *a* and *b* are the parameters.

### 2.13. Statistical Analysis

All experiments were conducted in triplicate minimum. The experimental data from both loading and release studies were subjected to curve fitting analysis using Origin software (Origin 2024). For the statistical analysis, we used SPSS 23 (one-way ANOVA), with *p* < 0.05 considered statistically significant.

## 3. Results

### 3.1. Physicochemical Characterizations of Gel/Alg-CMCS-Based Cationic Coordination Hydrogels

**Figure 1:**
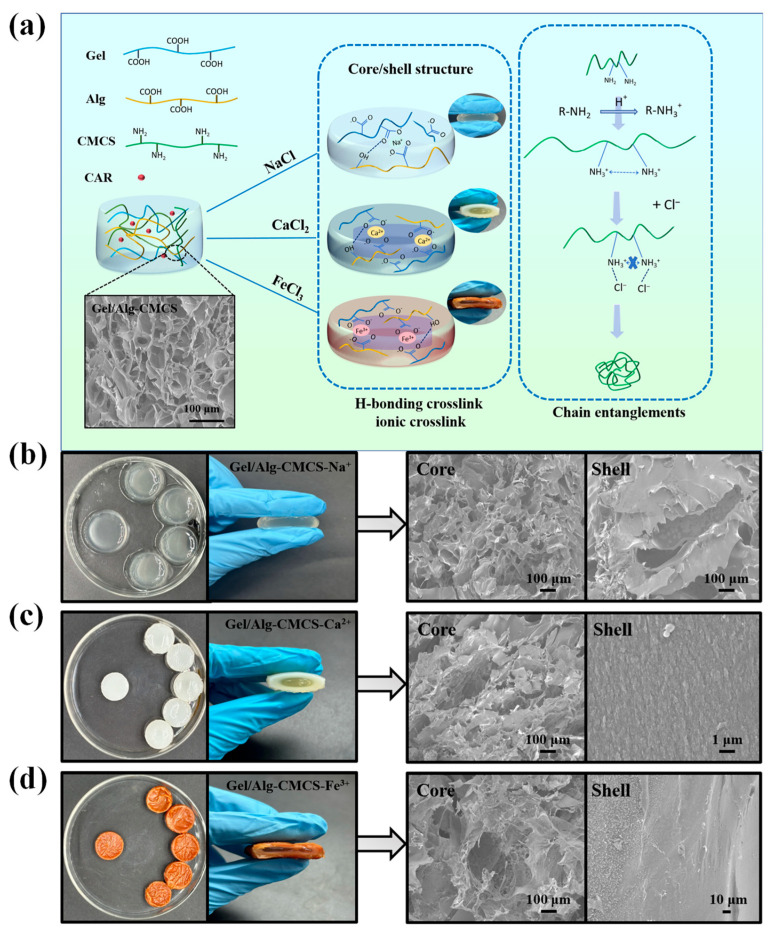
(**a**) Synthesis mechanism of Gel/Alg-CMCS-based cationic coordination hydrogels, digital photographs, and SEM of the core and shell of (**b**) Gel/Alg-CMCS-Na^+^, (**c**) Gel/Alg-CMCS-Ca^2+^, and (**d**) Gel/Alg-CMCS-Fe^3+^ hydrogel.

The pH-responsive Gel/Alg-CMCS hydrogels construct a three-dimensional network structure through polyelectrolyte interactions. The anions (-COO^−^) formed by the ionization of carboxyl groups (-COOH) on Gel and Alg undergo ionic crosslinking with cations (-NH_3_^+^) generated from the protonation of amino groups (-NH_2_) on CMCS under electrostatic attraction. This process forms a hydrogel with a dual crosslinked network structure and pH responsiveness. However, in highly hydrous polymer materials like hydrogels, an increase in the swelling ratio significantly degrades properties such as mechanical strength and self-healing behavior, severely limiting their applications. Therefore, effective control of the swelling ratio is crucial not only for ensuring stable performance but also for practical utility. When the Gel/Alg-CMCS hydrogel is immersed in a metal salt solution, the high osmotic pressure established between the hydrogel and the solution causes the rapid migration of internal water to the external medium, leading to hydrogel contraction [21]. Concurrently, metal cations and chloride ions from the salt solution slowly migrate into the hydrogel, interacting with the carboxyl groups of Alg and the amino groups of CMCS, respectively, thereby forming a chain entanglement network and an ionic crosslinked network; the resulting electrostatic repulsion effect induces hydrogel reswelling, while the free water inside the hydrogel continuously migrates outward, forming a rigid layer containing only bound water (Figure 1a). Due to the slow diffusion of the metal salt solution from the hydrogel surface toward the interior, the coordination between -COO^−^ and metal cations exhibits a gradient distribution. Rapid dehydration at the hydrogel surface increases the density of the polymer network, facilitating the formation of cationic-coordinated Gel/Alg-CMCS hydrogels with a soft core/hard shell structure. To better understand the impact of cations on the hydrogels, we prepared three metal salt solutions (0.1 M NaCl, CaCl_2_, FeCl_3_) to treat the hydrogels via immersion for 6 h, yielding Gel/Alg-CMCS-Na^+^ > hydrogels with a weak core–shell structure and Gel/Alg-CMCS-Ca^2+^ as well as Gel/Alg-CMCS-Fe^3+^ hydrogels with rigid core–shell structures.

SEM images (Figure 1a) demonstrate that the Gel/Alg-CMCS hydrogel prepared through polyelectrolyte interactions exhibits a three-dimensional network structure with thickened pore walls and non-uniformly distributed pore sizes, which contribute to higher tensile strength by promoting better stress dispersion, endowing the hydrogel with excellent mechanical properties while enabling the effective loading of CAR. Regarding the SEM characterization of Gel/Alg-CMCS-Na^+^, Gel/Alg-CMCS-Ca^2+^, and Gel/Alg-CMCS-Fe^3+^ hydrogels, the Gel/Alg-CMCS-Na^+^ hydrogel displays no hard shell structure but shows an inhomogeneous network distribution at its periphery compared to the interior (Figure 1b), indicating weak aggregation of monovalent cations and weak crosslinking with carboxyl groups, resulting in a feeble core–shell structure incapable of forming a rigid shell. Figure 1c,d illustrate that Gel/Alg-CMCS-Ca^2+^ and Gel/Alg-CMCS-Fe^3+^ hydrogels possess rigid core–shell structures, where the soft core features a loose network analogous to soft extracellular matrix in biological tissues for maintaining structural integrity and providing toughness, while the hard shell comprises a densely crosslinked network resembling the compact collagen fibers in biological tissues that confer mechanical strength [16]. The primary driving force for rigid shell formation primarily arises from osmotic pressure and ion migration; higher osmotic pressure causes rapid dehydration at the hydrogel surface, forming a rigid outer layer containing only bound water, which enhances the polymer network density. Simultaneously, metal ions diffuse from the surrounding solution into the hydrogel matrix and interact with carboxyl groups. Due to gradient diffusion behavior, a densely crosslinked polymer network forms and propagates radially inward from the surface. Furthermore, the hard shell microstructure of Gel/Alg-CMCS-Fe^3+^ hydrogel becomes more compact and uniformly distributed compared to that of Gel/Alg-CMCS-Ca^2+^, attributed to the higher crosslinking density of trivalent iron ions with carboxyl groups than divalent calcium ions, ultimately resulting in greater rigidity and smaller porosity.

**Figure 2:**
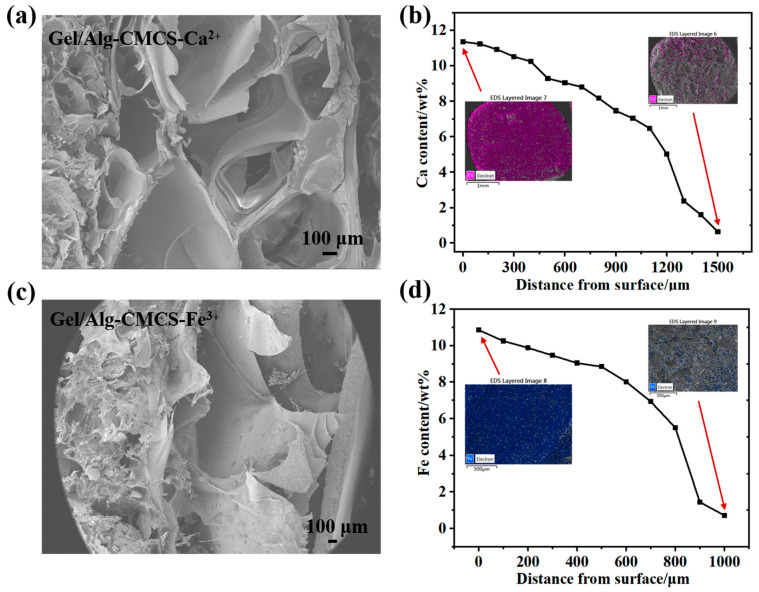
Shell thickness distribution of the cross-section of (**a**) Gel/Alg-CMCS-Ca^2+^ and (**c**) Gel/Alg-CMCS-Fe^3+^ hydrogels; EDX scanning of (**b**) Ca^2+^ concentration gradient in Gel/Alg-CMCS-Ca^2+^ hydrogel and (**d**) Fe^3+^ concentration gradient in Gel/Alg-CMCS-Fe^3+^ hydrogel.

Figure 2a,c exhibit the cross-sectional morphology and shell layer distribution of Gel/Alg-CMCS-Fe^3+^ and Gel/Alg-CMCS-Ca^2+^ hydrogels. These hydrogels possessed a dual-layer structure comprising a rigid shell and a soft core, with a porous intermediate region primarily resulting from the volume contraction during the phase transition. Due to its higher charge and coordination capacity, Fe^3+^ generated a thick and uniform shell, whereas Ca^2+^ formed a thin surface-concentrated layer. As the outer layer of the hydrogel initially contacts, the metal salt solution and metal ions gradually penetrate inward to form coordination bonds with COO^−^, and the metal ion concentration decreases progressively from the shell to the core. The Fe^3+^ content decreased from 10.86% to 0.72% (Figure 2b), and the Ca^2+^ content declined from 11.36% to 0.63% (Figure 2d).

**Figure 3:**
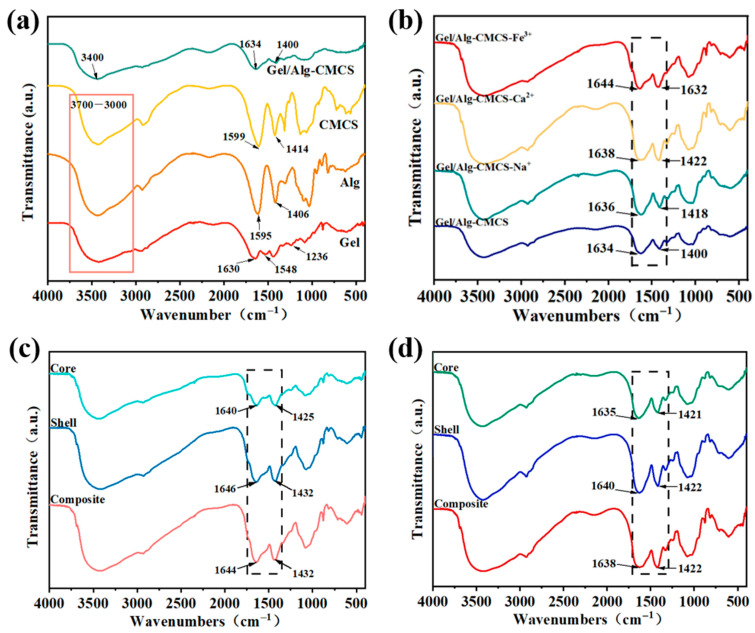
FTIR spectra of (**a**) Gel/Alg-CMCS hydrogel, (**b**) Gel/Alg-CMCS-based cationic coordination hydrogels, (**c**) Gel/Alg-CMCS-Ca^2+^ hydrogel, and (**d**) Gel/Alg-CMCS-Fe^3+^ hydrogel.

The FTIR spectra of Gel, Alg, CMCS, and Gel/Alg-CMCS samples are presented in Figure 3a. For Gel, the bands at 1630, 1548, and 1236 cm^−1^ correspond to the absorption of amide I, amide II, and amide III, respectively, while the broad peaks between 3700 and 3000 cm^−1^ are attributed to the stretching vibrations of N-H and O-H [22]. In the Alg spectrum, the broad peaks between 3700 and 3000 cm^−1^ correspond to the stretching vibration of O-H. Two characteristic peaks at 1595 and 1406 cm^−1^ are associated with the asymmetric and symmetric stretching vibrations of -COO^−^ [23]. In the CMCS spectrum, a broad absorption peak at 3700–3000 cm^−1^ is attributed to the overlapping stretching vibrations of the O-H group and the N-H bond of the primary amine group. The characteristic peaks at 1601 and 1414 cm^−1^ correspond to the asymmetric and symmetric stretching vibrations of the carboxylate anion [24]. Notably, the symmetric COO^−^ stretching vibration at 1601 cm^−1^ overlaps with the N-H bending vibration [25,26].

With the incorporation of CMCS, the characteristic peaks of -COO^−^ in Alg shifted from 1595 and 1406 cm^−1^ to 1634 and 1400 cm^−1^, respectively. Additionally, a significant blue-shifting and reduction in the peak intensity of the O-H stretching band (around 3400 cm^−1^) in Gel/Alg-CMCS hydrogels indicated the formation of intermolecular hydrogen bonds among Gel, Alg, and CMCS. Similar findings have been reported for other chitosan and sodium alginate-based hydrogels [27,28]. The peak intensities of NH_2_ characteristic peaks in CMCS at 3400 cm^−1^ decreased significantly, likely due to the formation of hydrogels through interactions between the NH_3_^+^ groups of CMCS and the COO^−^ groups of Alg [29]. In the spectrum of Gel/Alg-CMCS, characteristic peaks at 1634 and 1400 cm^−1^ belong to the asymmetric and symmetric stretching vibrations of -COO^−^. As observed in the FTIR spectra of Figure 3b, the characteristic peaks of COO^−^ in Gel/Alg-CMCS-Na^+^ shifted from 1634 and 1400 cm^−1^ to 1636 and 1418 cm^−1^, respectively, with the peak intensity ratio increasing from 0.7721 (I_1634_/I_1400_) to 0.8237 (I_1636_/I_1422_). In Gel/Alg-CMCS-Ca^2+^, the characteristic peaks of COO^−^ blue-shifted to 1638 and 1422 cm^−1^, with a peak intensity ratio of 0.850 (I_1638_/I_1422_). For Gel/Alg-CMCS-Fe^3+^, the characteristic peaks of COO^−^ further blue-shifted to 1644 and 1432 cm^−1^, and the peak intensity ratio reached 0.9780 (I_1636_/I_1432_). With increasing charge numbers of metal cations, the stretching intensity of the -COO^−^ characteristic peaks gradually decreased, and the COO^−^ absorption peaks shifted toward higher wavenumbers. This indicates that metal ions with higher charges interact with more carboxyl groups on Alg chains, resulting in higher coordination numbers, stronger coordination bonds, and tighter structures. Furthermore, compared to Ca^2+^ and Na^+^, Fe^3+^ cations with higher charges can preferentially occupy larger interstitial spaces within the Gel/Alg-CMCS hydrogels, forming more compact structures. As shown in the FTIR spectra of Figure 3c,d, the stretching intensity of the -COO^−^ characteristic peaks in the hard shell of Gel/Alg-CMCS-Ca^2+^ and Gel/Alg-CMCS-Fe^3+^ hydrogels is significantly lower than that in the soft core structures. For Gel/Alg-CMCS-Ca^2+^ hydrogel, the COO^−^ peak intensity ratio increased from 0.8431 (I_1633_/I_1421_) in the soft core to 0.8620 (I_1638_/I_1422_) in the hard shell. Similarly, for Gel/Alg-CMCS-Fe^3+^ hydrogel, the COO^−^ peak intensity ratio increased from 0.9260 (I_1640_/I_1425_) in the soft core to 1.004 (I_1646_/I_1432_) in the hard shell, indicating strong interactions between -COO^−^ and metal ions [30]. This phenomenon is attributed to the slow diffusion of chloride salt solution from the hydrogel surface toward the interior, where more metal ions bind with -COO^−^ at the surface to form the hard shell structure.

### 3.2. Mechanical Properties of Anti-Swelling Hydrogels

**Figure 4:**
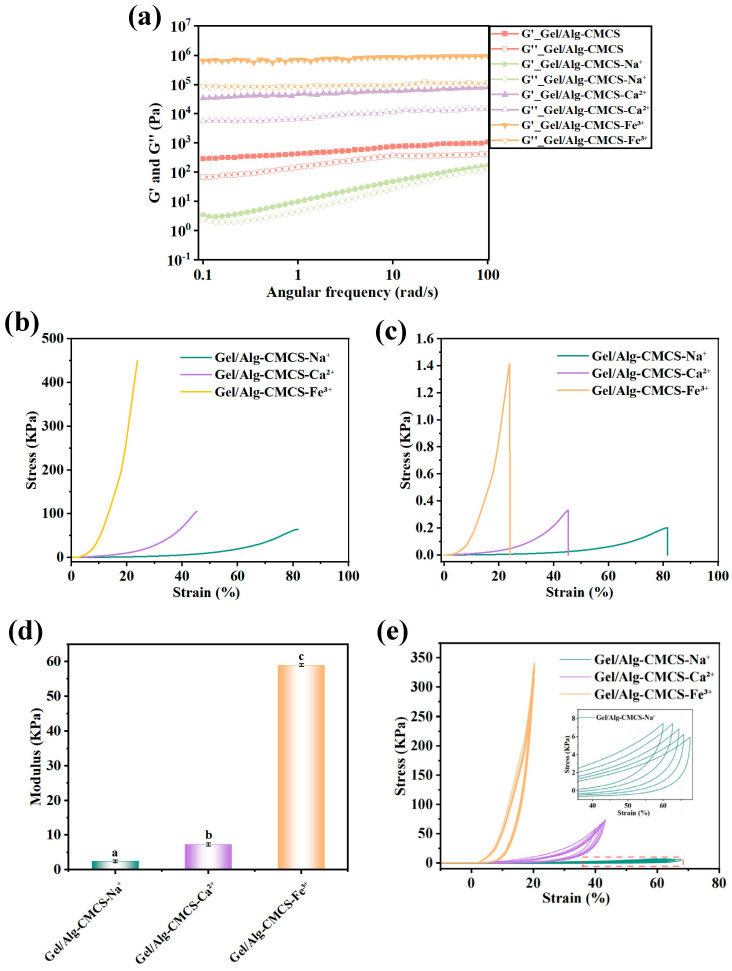
(**a**) G′ and G″ versus angular frequency in the measurement of rheology of Gel/Alg-CMCS hydrogel. (**b**) Compressive strength, (**c**) compressive stress–strain curves, (**d**) Young’s modulus of the hydrogels, and (**e**) loading–unloading tests of the hydrogels with varied compressive strains. Note: Different lowercase letters (a, b, c) denote statistically significant differences in the hydrogels (*p* < 0.05). The error bars indicate the standard deviation of *n* = 3.

The rheological properties of Gel/Alg-CMCS hydrogels were evaluated, as shown in Figure 4a. Within the tested frequency range, the storage modulus (G′) of all Gel/Alg-CMCS hydrogels exceeded the corresponding loss modulus (G″). This behavior is characteristic of hydrogels, indicating a well-structured hydrogel network. As the Gel/Alg-CMCS-Na^+^ hydrogel did not form a core–shell structure, both its storage modulus (G′) and loss modulus (G″) decreased accordingly. In contrast, the storage modulus (G′) and loss modulus (G″) of Gel/Alg-CMCS-Fe^3+^ and Gel/Alg-CMCS-Ca^2+^ hydrogels with core–shell structures were significantly enhanced.

As depicted in Figure 4b–d, under 25.04% strain, the Gel/Alg-CMCS-Fe^3+^ hydrogel exhibited the highest compressive stress, reaching 1.42 kPa, with a compressive strength of 466.57 kPa. The Gel/Alg-CMCS-Ca^2+^ hydrogel followed, showing a compressive stress of 0.328 kPa and a compressive strength of 101.32 kPa at 49.98% strain. The Young’s modulus of the Gel/Alg-CMCS-Fe^3+^ hydrogel was 59.01 kPa, far exceeding those of Gel/Alg-CMCS-Ca^2+^ and Gel/Alg-CMCS-Na^+^ hydrogels. This confirms that the Gel/Alg-CMCS-Fe^3+^ hydrogel possesses the densest shell structure and the best mechanical performance. Figure 4e displays the cyclic compression loading–unloading results of Gel/Alg-CMCS-Fe^3+^, Gel/Alg-CMCS-Ca^2+^, and Gel/Alg-CMCS-Na^+^ hydrogels over five cycles at 20%, 40%, and 60% compressive strains, respectively. Gel/Alg-CMCS-Na^+^ exhibited the lowest stress levels across all strain ranges (20–60%), attributed to weak ionic coordination (Na^+^ with COO^−^) that allows for rapid chain reorientation and energy dissipation, enabling excellent recovery after each cycle and demonstrating superior cyclic resilience. Gel/Alg-CMCS-Ca^2+^ hydrogel showed intermediate stress levels with moderate curve overlap across cycles. Divalent Ca^2+^ forms stronger coordination than Na^+^, providing greater structural stability, resulting in acceptable resilience with minor hysteresis. For Gel/Alg-CMCS-Fe^3+^ hydrogel, the curves from five cycles are concentrated and overlap closely without observable hysteresis, exhibiting typical elastic behavior. These results indicate that the cation-based anti-swelling hydrogels possess favorable cyclic resilience.

### 3.3. Anti-Swelling Behavior of Gel/Alg-CMCS-Based Cationic Coordination Hydrogels

**Figure 5:**
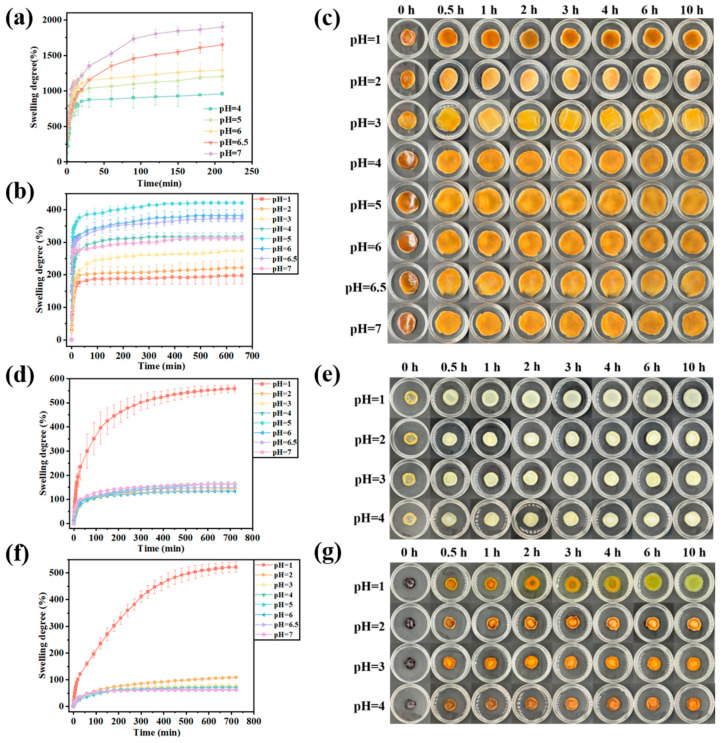
Swelling degree of (**a**) Gel/Alg-CMCS, (**b**) Gel/Alg-CMCS-Na^+^, (**d**) Gel/Alg-CMCS-Ca^2+^, and (**f**) Gel/Alg-CMCS-Fe^3+^ hydrogels and digital photographs of (**c**) Gel/Alg-CMCS-Na^+^, (**e**) Gel/Alg-CMCS-Ca^2+^, and (**g**) Gel/Alg-CMCS-Fe^3+^ hydrogels in different pH solutions. Note: The error bars indicate the standard deviation of *n* = 3.

The swelling curves of Gel/Alg-CMCS hydrogels at different pH values are presented in Figure 5a. As the pH increased, the swelling rate of Gel/Alg-CMCS hydrogels exhibited an upward trend. After 210 min, the swelling rate of Gel/Alg-CMCS hydrogels increased progressively with rising pH, reaching 958.32% at pH 4.0, 1202.56% at pH 5.0, 1293.48% at pH 6.0, 1649.87% at pH 6.5, and 1901.41% at pH 7.0. Under acidic conditions (pH 4.0), the -NH_2_ groups in CMCS are protonated into NH_3_^+^, and the -COO^−^ groups in Alg react with H^+^ ions in the solution to form -COOH. This weakens the polyelectrolyte interactions with CMCS, while hydrogen bonds form between -COOH and CMCS. The hydrogen bonding with water molecules is weakened, inhibiting the penetration of external water molecules and reducing the hydrogel’s swelling rate. Under neutral or slightly alkaline conditions, the -COOH groups in Gel and Alg primarily exist as -COO^−^ anions. The degree of -COOH dissociation increases with pH, leading to stronger electrostatic repulsion between -COO^−^ groups and facilitating the entry of water molecules into the hydrogels, thereby increasing the swelling rate.

Swelling tests were also conducted on Gel/Alg-CMCS-Na^+^, Gel/Alg-CMCS-Ca^2+^, and Gel/Alg-CMCS-Fe^3+^ hydrogels, and their swelling behaviors under different pH conditions are illustrated in Figure 5b–g. The results reveal that the swelling ratios of hydrogels significantly decreased after immersion in salt solutions. As Gel/Alg-CMCS-Na^+^ hydrogel lacked a rigid shell structure, its swelling behavior differed from that of Gel/Alg-CMCS-Ca^2+^ and Gel/Alg-CMCS-Fe^3+^ hydrogels. Specifically, for Gel/Alg-CMCS-Na^+^ hydrogel (Figure 5b), the swelling ratio gradually increased with decreasing pH in solutions above pH 4.0, reaching a maximum swelling ratio of 311.23% at pH 5.0 by 720 min. This is attributed to the weak core–shell structure in Gel/Alg-CMCS-Na^+^, where lower pH promotes the increased binding of sodium salts to anions, dissolving the shell structure and allowing for greater swelling. Conversely, in solutions below pH 5.0, the swelling ratio decreased with further pH reduction, achieving a minimum swelling ratio of 197.23% at pH 1.0 by 720 min, as the feeble core–shell structure completely dissolved, restoring the original swelling behavior (Figure 5c).

For Gel/Alg-CMCS-Ca^2+^ and Gel/Alg-CMCS-Fe^3+^ hydrogels with core–shell structures (Figure 5d–g), the Gel/Alg-CMCS hydrogel exhibited swelling ratios ranging from 958.32% (pH 4.0) to 1901.41% (pH 7.0) at 210 min. At pH > 1.0, Gel/Alg-CMCS-Ca^2+^ showed a swelling ratio below 150% at 720 min, while Gel/Alg-CMCS-Fe^3+^ exhibited almost no swelling at 720 min. This difference stems from Fe^3+^ demonstrating the strongest coordination ability due to its high charge density and small ionic radius. The higher charge of trivalent iron ions compared to divalent calcium ions enables denser crosslinking with carboxyl groups, resulting in a highly compact shell structure in Gel/Alg-CMCS-Fe^3+^ that impedes water penetration. However, at pH 1.0, the swelling ratio increased dramatically: Gel/Alg-CMCS-Ca^2+^ reached 558.67%, while Gel/Alg-CMCS-Fe^3+^ reached 521.57% by 720 min. This significant reswelling is caused by the dissociation of hydrophobic associations and metal–ligand bond interactions within the network structure under strong acidic conditions [31].

As shown in Table 1, no significant changes in the swelling behavior of Gel/Alg-CMCS-Na^+^, Gel/Alg-CMCS-Ca^2+^, and Gel/Alg-CMCS-Fe^3+^ hydrogels were observed after 120 days of storage, further confirming their exceptional long-term anti-swelling stability.

### 3.4. Safety of Gel/Alg-CMCS-Based Cationic Coordination Hydrogels

**Figure 6:**
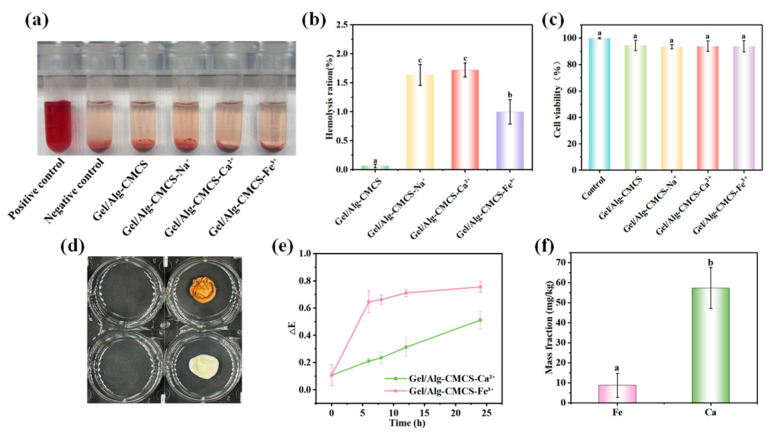
(**a**) Changes observed after centrifugation of Gel/Alg-CMCS-based cationic coordination hydrogel incubated with red blood cells for 1 h and (**b**) hemolysis rate. (**c**) Cell viability of Gel/Alg-CMCS-based cationic coordination hydrogels. (**d**) Photographs and (**e**) ΔE changes in the food simulant after Gel/Alg-CMCS-Ca^2+^ and Gel/Alg-CMCS-Fe^3+^ hydrogels were immersed for 24 h. (**f**) Mass fractions of ions’ migration from hydrogels to medium. Note: Different lowercase letters (a, b, c) denote statistically significant differences in the hydrogels (*p* < 0.05). The error bars indicate the standard deviation of *n* = 3.

As shown in Figure 6a,b, none of the cationic anti-swelling hydrogels exhibited hemolytic activity. The hemolysis rates of all hydrogel samples were lower than 2%, significantly below the commonly used 5% threshold in biological material safety standards. This indicates that the cationic anti-swelling hydrogels possess high safety profiles and cause minimal damage to red blood cells. Cytotoxicity assessment demonstrates good biocompatibility for all tested samples (Figure 6c), with cell viabilities of 94.51% (Gel/Alg-CMCS), 93.48% (Gel/Alg-CMCS-Na^+^), 93.84% (Gel/Alg-CMCS-Ca^2+^), and 93.82% (Gel/Alg-CMCS-Fe^3+^). The cell viabilities of all samples remained above 90%, exhibiting an approximately 7% reduction compared to the control group, indicating negligible toxic effects (conforming to ISO 10993-5) [32]. These cytotoxicity results confirm the favorable biosafety of Gel/Alg-CMCS-based cationic coordination hydrogels, supporting their potential applications in food-related fields.

As shown in Figure 6d, the food simulant remained clear and transparent after Gel/Alg-CMCS-Ca^2+^ and Gel/Alg-CMCS-Fe^3+^ hydrogels were immersed for 24 h. The color difference values of the medium were inferior to 0.8 (Figure 6e), falling below the human-perceptible color change threshold, demonstrating that the food matrix was scarcely stained by coming into contact with hydrogels. As shown in Figure 6f, after 24 h of immersion in the food simulant, the mass fraction of Fe^3+^ ions’ migration from the Gel/Alg-CMCS-Fe^3+^ hydrogel was 8.82 mg/kg, and that of Ca^2+^ ions was 57.31 mg/kg. According to the Chinese national standard GB 9685-2016 (Standard for Uses of Additives in Food Contact Materials and Products) [33], the migration limit of Fe^3+^ ions should be less than 48 mg/kg, while no specific requirement is set for Ca^2+^ ions. Thus, the residual free ions in the food simulant remain within the regulatory limits for the intended applications.

### 3.5. Loading Efficiency of Gel/Alg-CMCS-Based Cationic Coordination Hydrogels

**Figure 7:**
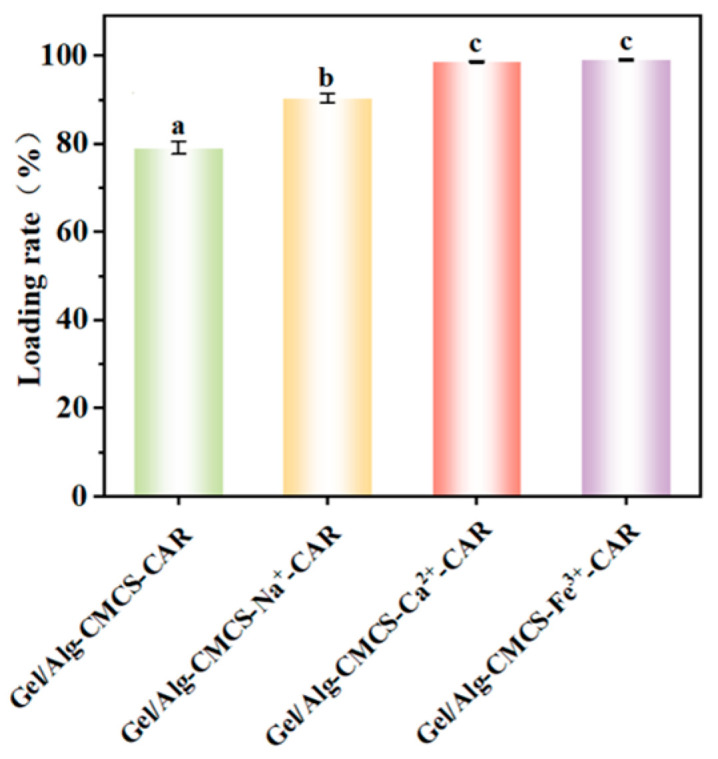
Loading rate of Gel/Alg-CMCS-based cationic coordination-CAR hydrogels. Note: Different lowercase letters (a, b, c) denote statistically significant differences in the hydrogels (*p* < 0.05). The error bars indicate the standard deviation of *n* = 3.

The loading of CAR in Gel/Alg-CMCS-based cationic coordination hydrogels is shown in Figure 7, revealing that compared with Gel/Alg-CMCS-CAR hydrogel, the CAR loading capacity of Gel/Alg-CMCS-based cationic coordination-CAR hydrogels is significantly enhanced. Specifically, the CAR loading rate of Gel/Alg-CMCS-Na^+^-CAR hydrogel increases from the original 79.06% to 90.28%, while Gel/Alg-CMCS-Ca^2+^-CAR and Gel/Alg-CMCS-Fe^3+^-CAR hydrogels with rigid core–shell structures achieve CAR loading rates of 98.63% and 99.07%, respectively.

### 3.6. CAR Release from Gel/Alg-CMCS-Based Cationic Coordination Hydrogels

**Figure 8:**
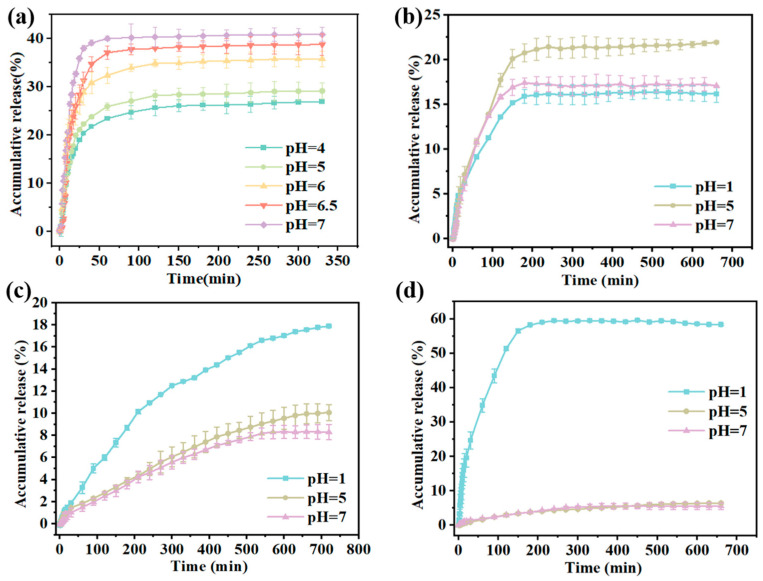
Cumulative release of (**a**) Gel/Alg-CMCS-CAR, (**b**) Gel/Alg-CMCS-Na^+^-CAR, (**c**) Gel/Alg-CMCS-Ca^2+^-CAR, and (**d**) Gel/Alg-CMCS-Fe^3+^-CAR hydrogels in different pH solutions. Note: The error bars indicate the standard deviation of *n* = 3.

The release of CAR from Gel/Alg-CMCS-CAR hydrogels under different pH conditions is illustrated in Figure 8a. The release of CAR from the hydrogels exhibited three distinct stages: an initial burst, sustained release, and a fixed release phase. As the buffer pH increased, the CAR release time of the hydrogels decreased. The release rate and cumulative release amount of the hydrogels increased with the pH of the buffer solution. After 330 min, the cumulative release of Gel/Alg-CMCS-CAR hydrogels was 26.96% at pH 4.0 and 40.83% at pH 7.0. Under acidic conditions, the -COO^−^ groups of Alg are converted to -COOH, reducing the degree of ionization and causing the Alg molecular chains to shrink, thereby densifying the hydrogel shell. However, as the pH increases, the degree of dissociation of -COOH groups rises, leading to stronger repulsion between -COO^−^ groups. This results in the expansion of the hydrogel. Thus, CAR release from the hydrogels is inhibited under acidic conditions compared to slightly alkaline conditions. Conversely, under alkaline conditions, the CAR release rate from the hydrogels increased.

The release profiles of CAR from Gel/Alg-CMCS-Na^+^-CAR, Gel/Alg-CMCS-Ca^2+^-CAR, and Gel/Alg-CMCS-Fe^3+^-CAR hydrogels under different pH conditions are presented in Figure 8b–d. At 660 min, the cumulative release of CAR from Gel/Alg-CMCS-Na^+^-CAR hydrogel reached 17.06% at pH 7.0 and 21.92% at pH 5.0, attributed to the dissolution of the weak shell structure resulting from the increased binding of sodium salts to anions, which releases hydrogel constraints and facilitates CAR release. At pH 1.0, the feeble core–shell structure of Gel/Alg-CMCS-Na^+^-CAR hydrogel was completely dissolved, exhibiting the original low-swelling behavior under acidic conditions, thereby reducing the cumulative CAR release to 16.16%. Under strongly acidic conditions (pH 1.0) at 720 min, the cumulative releases from Gel/Alg-CMCS-Ca^2+^-CAR and Gel/Alg-CMCS-Fe^3+^-CAR hydrogels were 17.88% and 58.34%, respectively. At pH 5.0 and 7.0, the cumulative releases from Gel/Alg-CMCS-Ca^2+^-CAR hydrogel remained below 10%, while those from Gel/Alg-CMCS-Fe^3+^-CAR hydrogel were below 7%. Consistent with the swelling behavior, these results indicate that higher CAR release under acidic conditions (pH 1.0) and lower release at pH 5.0 and 7.0 are governed by hydrogel swelling: higher swelling ratios correspond to relatively looser network structures and larger pore sizes, enabling CAR release, whereas lower swelling impedes release.

**Figure 9:**
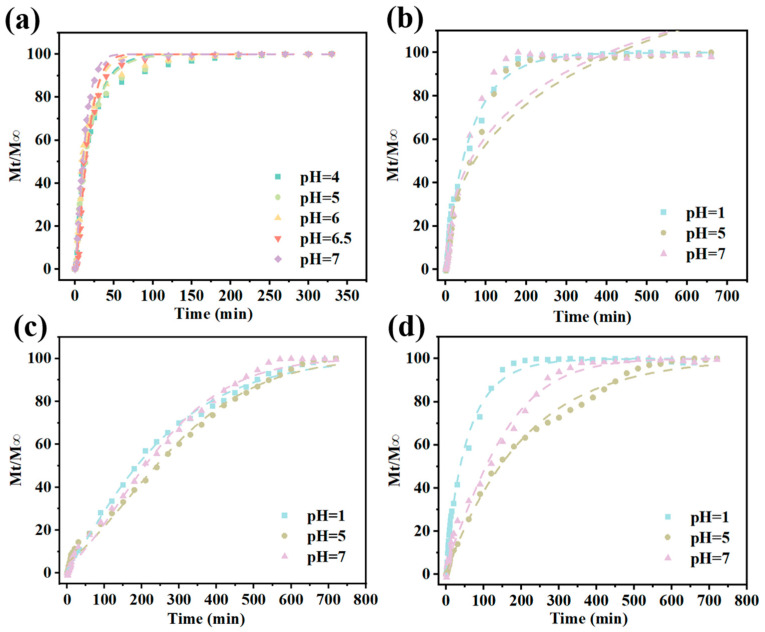
Weibull fitting of (**a**) Gel/Alg-CMCS-CAR, (**b**) Gel/Alg-CMCS- Na^+^-CAR, (**c**) Gel/Alg-CMCS-Ca^2+^-CAR, and (**d**) Gel/Alg-CMCS-Fe^3+^-CAR hydrogels in different pH solutions.

To further elucidate the release mechanisms, the release profiles of CAR from cation-based Gel/Alg-CMCS-CAR hydrogels in different pH solutions were analyzed using the Higuchi, Korsmeyer–Peppas, Peppas–Sahlin, and Weibull models (Table 2). The model that best described the CAR release behavior was selected based on the highest correlation coefficient (R^2^ > 0.9). Compared to other kinetic models, the Weibull model was the most suitable for characterizing the release kinetics (Figure 9a–d). The parameter *b* in the Weibull model is the shape parameter and the most critical one in the entire model. When *b* ≤ 0.75, the drug diffusion type is Fickian diffusion; when 0.75 < *b* < 1.0, the drug diffusion type is mixed diffusion, where both diffusion and polymer chain relaxation/erosion contribute, but diffusion remains dominant; and when *b* ≥ 1.0, the release mechanism is complex, indicating that the release is not purely diffusion-based and may include Case-II relaxation, erosion control, and open-form dosage forms. As shown in Table 3, the release of CAR from Gel/Alg-CMCS-CAR hydrogel is primarily erosion-dominated, with diffusion as a secondary mechanism. The Gel/Alg-CMCS-Na^+^-CAR hydrogel, which lacks a core–shell structure and has a loose polymer network, exhibits release behavior close to combined diffusion/erosion at pH 1.0, while Fickian diffusion dominates at pH 5.0 or 7.0. The Gel/Alg-CMCS-Ca^2+^-CAR hydrogel demonstrates erosion/polymer relaxation-dominated release under all tested pH conditions, attributed to the Ca^2+^-induced dense shell delaying diffusion. For the Gel/Alg-CMCS-Fe^3+^-CAR hydrogel, the strong acid at pH 1.0 induces shell collapse, resulting in Fickian diffusion as the release mechanism. Under pH 5.0 or 7.0 conditions, the release is erosion-dominated due to the stable coordination bond structure formed by Fe^3+^.

## 4. Conclusions

In this work, the polyelectrolyte Gel/Alg-CMCS hydrogel was fabricated and exhibited good pH-responsive CAR release behavior. Via the incorporation of positive ion, such as Na^+^, Ca^2+^, and Fe^3+^, the hydrogel exhibited a unique shell–core structure. The Gel/Alg-CMCS-Fe^3+^ hydrogel exhibited the highest mechanical performance, with a compressive strength reaching 466.57 KPa. The thickness of the shell structure was enhanced with the increase in cation valence. The cationic coordination hydrogel possessed different anti-swelling properties. With the thickest shell, the Gel/Alg-CMCS-Fe^3+^ hydrogel rarely absorbed water at pH 2.0–7.0, while it swelled slowly to 521.57% via shell collapse at pH 1.0. Meanwhile, the intrinsic pH-responsive release behavior of Gel/Alg-CMCS hydrogel, which resisted the release of CAR under acidic conditions but rapid release under neutral conditions, was converted by the participation of positive ion. In particular, the Gel/Alg-CMCS-Fe^3+^ hydrogel demonstrated high accumulative release of 58.34% at pH 1.0, while it showed a low release degree of only 7% in weakly acidic and neutral environments. Thus, the anti-swelling Gel/Alg-CMCS-based cationic coordination hydrogels were proposed as pH-responsive delivery systems for application in the food industry. However, the relatively high cost of Fe^3+^ in raw materials and the rust-red color exhibited by the hydrogel may affect consumer acceptance. Future research should focus on identifying alternative materials with lower costs and improved sensory attributes.

## Figures and Tables

**Table 1:** Swelling behavior of Gel/Alg-CMCS-Na^+^, Gel/Alg-CMCS-Ca^2+^, and Gel/Alg-CMCS-Fe^3+^ hydrogels in different pH environments after 120 days of storage.

Sample	pH	0 d	120 d
Gel/Alg-CMCS-Na^+^	1.0	197.23 ± 25.66% ^a^	192.81 ± 20.77% ^a^
	5.0	421.21 ± 5.05% ^a^	417.16 ± 2.92% ^a^
	7.0	311.23 ± 7.04% ^a^	307.26 ± 6.19% ^a^
Gel/Alg-CMCS-Ca^2+^	1.0	556.06 ± 19.21% ^a^	550.28 ± 15.45% ^a^
	5.0	161.85 ± 2.49% ^a^	157.73 ± 1.45% ^a^
	7.0	163.30 ± 1.95% ^a^	156.62 ± 1.62% ^a^
Gel/Alg-CMCS-Fe^3+^	1.0	518.38 ± 20.27% ^a^	513.50 ± 11.22% ^a^
	5.0	64.66 ± 2.49% ^a^	60.54 ± 1.05% ^a^
	7.0	61.42 ± 0.61% ^a^	59.34 ± 0.64% ^a^

Note: Different lowercase letters (adenote statistically significant differences in the swelling rates of hydrogels at 0 d and 120 d (*p* < 0.05). The error bars indicate the standard deviation of *n* = 3.

**Table 2:** The correlation coefficient (R^2^) of the Higuchi, Korsmeyer–Peppas, Peppas–Sahlin, and Weibull models.

Sample	pH	R^2^			
		Higuchi Model	Korsmeyer–Peppas Model	Peppas–Sahlin Model	Weibull Model
Gel/Alg-CMCS-CAR	4.0	0.6239	0.7167	0.9410	0.9833
	5.0	0.5854	0.6953	−5.9632	0.9928
	6.0	0.4757	0.6421	−5.8949	0.9796
	6.5	0.6359	0.6867	−4.4557	0.9853
	7.0	0.3653	0.6269	−5.6786	0.9968
Gel/Alg-CMCS-Na^+^-CAR	1.0	0.8473	0.8541	0.9867	0.9956
	5.0	0.8885	0.8912	0.9284	0.9377
	7.0	0.8614	0.8575	0.9106	0.9194
Gel/Alg-CMCS-Ca^2+^-CAR	1.0	0.9798	0.9504	0.9952	0.9977
	5.0	0.9744	0.9380	0.9933	0.9948
	7.0	0.9685	0.9343	0.9912	0.9960
Gel/Alg-CMCS-Fe^3+^-CAR	1.0	0.8344	0.7506	0.9889	0.9965
	5.0	0.9849	0.9231	0.9955	0.9960
	7.0	0.9628	0.9720	0.9792	0.9945

**Table 3:** The kinetic parameters of the Weibull model.

Sample	pH	R^2^	b
Gel/Alg-CMCS-CAR	4.0	0.9833	1.0029 ± 0.0921
	5.0	0.9928	1.0199 ± 0.0369
	6.0	0.9796	1.0393 ± 0.1245
	6.5	0.9853	1.3592 ± 0.1987
	7.0	0.9968	1.2785 ± 0.0792
Gel/Alg-CMCS-Na^+^-CAR	1.0	0.9956	0.9027 ± 0.0270
	5.0	0.9284	0.4999 ± 0.1006
	7.0	0.9106	0.4998 ± 0.1061
Gel/Alg-CMCS-Ca^2+^-CAR	1.0	0.9977	1.2075 ± 0.0380
	5.0	0.9948	1.7150 ± 0.1158
	7.0	0.9960	1.6359 ± 0.0945
Gel/Alg-CMCS-Fe^3+^-CAR	1.0	0.9965	0.8700 ± 0.0224
	5.0	0.9960	1.0221 ± 0.0400
	7.0	0.9945	1.2796 ± 0.0989

## Data Availability

The original contributions presented in the study are included in the article, further inquiries can be directed to the corresponding author.
